# Assembly and Valence Modulation of Ordered Bimetallic MOFs for Highly Efficient Electrocatalytic Water Oxidation

**DOI:** 10.3390/molecules29245845

**Published:** 2024-12-11

**Authors:** Yaling Wu, Zhaopeng Sun, Yingying Chen, Dan Liu, Yan Meng, Zheng Yan

**Affiliations:** 1 Anhui Provincial Key Laboratory of Advanced Catalysis and Energy Materials, Anqing Normal University, Anqing 246133, China; wuyaling2627@163.com; 2 College of Biological, Chemical Sciences and Engineering, Jiaxing University, Jiaxing 314001, China; sakura.189663@gmail.com (Z.S.); chenyying0918@163.com (Y.C.); liudan@mail.zjxu.edu.cn (D.L.)

**Keywords:** template-directed, bimetallic organic frameworks, oxygen evolution reaction, valence state regulated

## Abstract

Metal synergy can enhance the catalytic performance, and a prefabricated solid precursor can guide the ordered embedding, of secondary metal source ions for the rapid synthesis of bimetallic organic frameworks (MM’-MOFs) with a stoichiometric ratio of 1:1. In this paper, **Co-MOF-1D** containing well-defined binding sites was synthesized by mechanical ball milling, which was used as a template for the induced introduction of Fe ions to successfully assemble the ordered bimetallic **Co_1_Fe_1_-MOF-74@2** (where **@2** denotes template-directed synthesis of MOF-74). Its electrocatalytic performance is superior to that of the conventional one-step-synthesized **Co_1_Fe_1_-MOF-74@1** (where **@1** denotes one-step synthesis of MOF-74), and the ratio of the two metal sources, Co and Fe, is close to 1:1. Meanwhile, the iron valence states (Fe^II^ and Fe^III^) in **Co_1_Fe_1_-MOF-74@2** were further regulated to obtain the electrocatalytic materials **Co_1_Fe_1_(II)-MOF-74@2** and **Co_1_Fe_1_(III)-MOF-74@2**. The electrochemical performance test results confirm that **Co_1_Fe_1_(II)-MOF-74@2** regulated by valence state has a better catalytic performance than **Co_1_Fe_1_(III)-MOF-74@2** in the oxygen evolution reaction (OER) process. This phenomenon is related to the gradual increase in the valence state of Fe ions in **Co_1_Fe_1_(II)-MOF-74@2**, which promotes the continuous improvement in the performance of the MOF before reaching the optimal steady state and makes the OER performance reach the optimum when the Fe^II^/Fe^III^ mixed-valence state reaches a certain proportion. This provides a new idea for the directed synthesis and optimization of highly efficient catalysts.

## 1. Introduction

Against the backdrop of energy crises and environmental degradation, researchers in the field of green chemistry are actively exploring new energy sources to replace fossil fuels to achieve the goal of carbon neutrality [1,2,3,4]. From a chemical point of view, the decomposition of water molecules as energy carriers into hydrogen, an effective way of energy conversion, is in line with the concept of sustainable development [5,6,7,8,9,10]. However, to achieve efficient conversion, it is necessary to overcome the problems existing in the sluggish kinetics and complex reaction pathways of the hydrogen evolution reaction (HER) and the oxygen evolution reaction (OER) [11,12,13,14,15,16,17]. Differing from traditional electrocatalysts containing precious metal elements such as platinum, ruthenium, iridium, etc., optimized electrocatalysts made of non-noble inorganic materials exhibit extraordinary intrinsic activity, stability, elemental abundance, and cost-controllable advantages, which make them the key to promoting the widespread application of new energy storage and conversion, and also the current research difficulty in the field of energy chemistry [18,19]. With various microstructure strategies (including reducing the size of nanostructures, generating vacancies, doping or alloying, and crystal-phase engineering [20,21,22,23,24]), the electronic structure of the catalyst can be effectively modulated, thereby influencing the bonding strength between the active sites and reactants in the processes of adsorption and desorption [25]. Doping is a commonly used microstructure strategy. By altering the proportion of elements within the catalyst, it has an impact on properties like electrical conductivity, electronegativity, and electronic energy [26,27]. This regulatory strategy can, in many instances, enhance the catalytic reactivity, particularly in reactions such as hydrogenation, oxidation, and cracking [28,29,30,31,32].

Mixed-metal MOFs (MM’-MOFs) are crystalline porous materials synthesized on the basis of MOFs by linking two or more different metal ions (usually metal centers) and organic ligands through coordination bonds [33]. MOF-74 has open metal sites, and during the synthesis process, various metal ions can be flexibly doped as needed. Recent research has also revealed that partially embedding different metal nodes into the MOF-74 framework can improve the adsorption performance, stability, and catalytic efficiency of MOF-74, and such performance enhancement is mainly attributed to the synergy among different metals [34,35,36]. In previous studies, Co and Fe have already demonstrated a typical synergistic effect in enhancing catalytic performance [37,38,39]. The Co element can adjust the active center’s electronic structure and modify the adsorption energy of reaction intermediates, facilitating the key reaction steps. Meanwhile, the Fe element is outstanding in optimizing the reaction pathway. Its unique chemical properties drive the reaction to progress in a more favorable direction and curtail the incidence of side reactions. Specifically, Fe can form specific chemical bonds or local electron interactions with Co, jointly constructing an efficient catalytically active site, enhancing the adsorption and conversion capabilities of the substrate and improving the catalytic efficiency and selectivity of the entire catalyst system for the target reaction. Nevertheless, it remains highly challenging to precisely control the stoichiometry and distribution of their metal centers. In the past, most of the multi-metal MOF-74 synthesized by the one-step solvothermal method could not precisely control the chemical composition, and the metal distribution in the product was significantly different from the stoichiometric ratio of the corresponding reactants [40,41,42,43]. With continuous research and exploration, Coskun et al. were the first to reveal the mystery of the template-directed method [44] and successfully realized the orderly embedding of two metal sources within the bimetallic–organic framework. Furthermore, researchers like Ghada employed a method of combining a specially designed solid-state precursor with a secondary metal source to construct the MOF-74 structure [45].This strategy provides a significant improvement in controlling the 1:1 stoichiometric ratio of the two different metals in the MOF and allows for well-defined discrete or polymeric metal–organic complexes to be used as precursors through room-temperature mechanochemical ball milling reactions [46,47].

**Figure 1:**
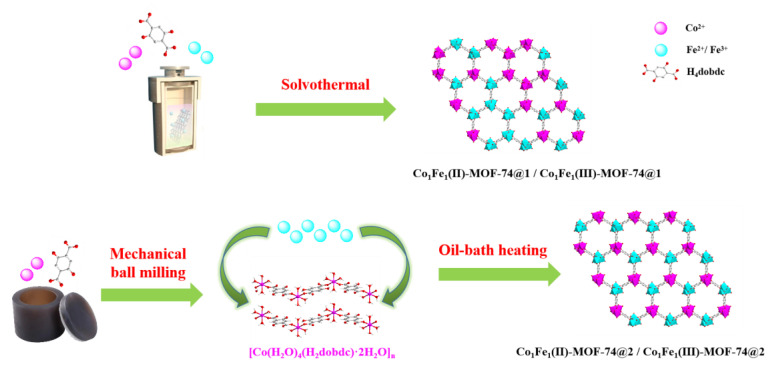
Schematic diagrams of the two synthetic process routes for the metal–organic framework Co_1_Fe_1_-MOF-74.

This paper adopts the template-directed synthesis strategy (Figure 1). Firstly, a one-dimensional cobalt-based metal–organic framework (**Co-MOF-1D**) with well-defined binding sites was synthesized by mechanical ball milling using Co^II^ and the organic ligand 2,5-dihydroxyterephthalic acid (H_4_dobdc) as raw materials. Using this structure as a template and Fe ions as a secondary metal source, the ordered bimetallic **Co_1_Fe_1_-MOF-74@2** was assembled, which showed better electrocatalytic performance than the conventional one-step-synthesized **Co_1_Fe_1_-MOF-74@1**, and the ratio of the two metal sources, Co and Fe, was close to 1:1. Meanwhile, this paper also targets the modulation of iron valence states (Fe^II^ and Fe^III^) in **Co_1_Fe_1_-MOF-74@2** to obtain the electrocatalytic materials **Co_1_Fe_1_(II)-MOF-74@2** and **Co_1_Fe_1_(III)-MOF-74@2**. The electrocatalytic OER performance showed that **Co_1_Fe_1_-MOF-74@2** electrocatalytic performance was superior to **Co_1_Fe_1_-MOF-74@1**; **Co_1_Fe_1_(II)-MOF-74@2** electrocatalytic performance was again superior to **Co_1_Fe_1_(III)-MOF-74@2**. This result confirms that assembly means, bimetallic synergism, and valence modulation collectively enhance the electrocatalytic performance of MOFs, which provides a research direction for targeted synthesis and optimization of efficient catalysts.

## 2. Results and Discussion

### 2.1. Structure and Morphology

**Figure 2:**
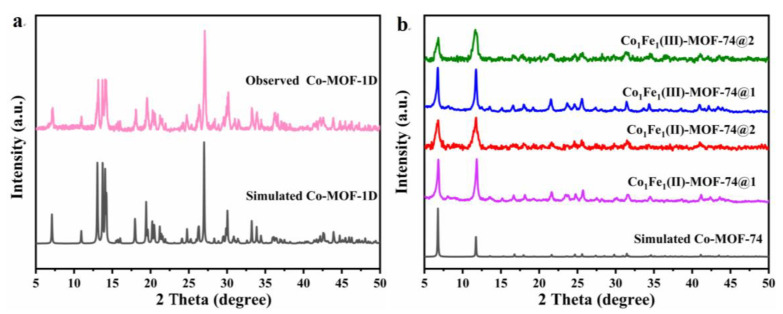
(**a**) XRD pattern of **Co-MOF-1D**; (**b**) XRD pattern of four MOFs.

In this paper, X-ray diffraction (XRD) is initially used to perform characterization of the physical phase of the resulting MOF samples. The results demonstrate that **Co-MOF-1D** is a crystalline material, and its XRD pattern is highly consistent with the simulated peak pattern (see Figure 2a), which confirms that **Co-MOF-1D** conforms to the [Co(H_2_O)_4_(H_2_dobdc)·2H_2_O]_n_ structure [48,49]. The formation of this structural system is due to π-π stacking interactions and hydrogen bonding interactions, and these forces contribute to the orderly alignment of the binding sites with the centers of the cobalt atoms, resulting in the formation of a specific sawtooth structure. **Co-MOF-1D** is synthesized via the coordination of the cobalt atom center with two carboxylic acid groups and water molecules. In this process, the hydroxyl and carbonyl moieties serve as well-defined binding sites, thereby facilitating the creation of conditions conducive to the ordered embedding of secondary metal source ions. Figure 2b shows the XRD comparison spectra of four MOF catalysts assembled by one-step synthesis and the template-directed method. As can be seen from this spectrum, these four MOFs catalysts have similar crystalline phases and their peak positions are highly compatible with the simulated Co-MOF-74, which conforms to the structure of [Co_2_(C_8_H_2_O_6_)(H_2_O)_2_·8H_2_O] with high purity. This result also indicates that different preparation methods and different valence iron ions have little effect on the overall structure of **Co_1_Fe_1_-MOF-74**. Furthermore, in light of the valence difference variations of iron ions, the **Co_1_Fe_1_(II)-MOF-74@2 (N_2_)** synthesized under a nitrogen atmosphere was investigated and contrasted with the **Co_1_Fe_1_(II)-MOF-74@2** prepared under normal circumstances via XRD analysis. It was found that the peak patterns of the two were highly congruent (e.g., Figure S1).

In addition, the Fourier transform infrared spectroscopy (FT-IR) characterization results (see Figure S2 for details) provide an important basis for an in-depth understanding of the structural properties of the four materials. It is clear from the FT-IR spectra that the four materials exhibit a high degree of similarity in the position and intensity of the absorption peaks in a given wave number range. This means that there is no significant difference between the four materials in terms of functional group composition. The thermogravimetric analysis (TGA) curves under air atmosphere (see Figure S3 for details) also lead to corresponding conclusions. Analysis of this thermogram shows that the decomposition processes of the four MOFs are carried out synchronously and that two different stages of mass reduction are displayed: before 500 K, there is a loss of about 15% of the mass, which is due to the removal of the physically uncoordinated guest molecules; however, after 500 K, the sharp mass drop is due to the removal of the organic ligands.

**Figure 3:**
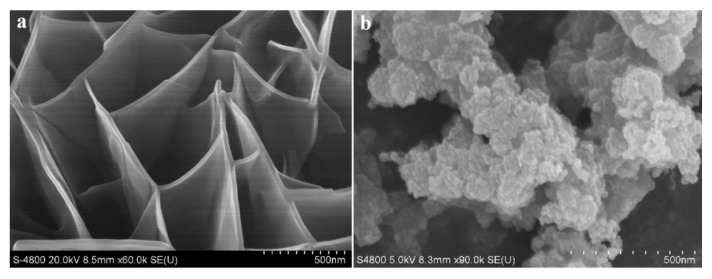
SEM images of (**a**) **Co-MOF-1D** and (**b**) **Co_1_Fe_1_(II)-MOF-74@2**.

With the help of the scanning electron microscopy (SEM) technique, the prepared **Co-MOF-1D** was characterized morphologically. As observed from the SEM images, **Co-MOF-1D** presents a honeycomb lamellar structure with nanometer thickness (Figure 3a). Using this structure as a template guide, the introduction of a secondary metal source, Fe ions, will make it easier to construct an ordered bimetallic **Co_1_Fe_1_-MOF-74**. Figure 3b and Figure S4 show that all four MOF catalysts (with a scale bar of 500 nm) are in the form of nanoparticles, and there is no significant difference in morphology. Further analysis of the SEM images shown in Figure S5 (with a scale bar of 100 nm) reveals that the average particle sizes of the four MOFs catalysts are as follows: **Co_1_Fe_1_(II)-MOF-74@1** (28.71 nm); (b) **Co_1_Fe_1_(II)-MOF-74@2** (26.33 nm); (c) **Co_1_Fe_1_(III)-MOF-74@1** (34.39 nm); and (d) **Co_1_Fe_1_(III)-MOF-74@2** (29.22 nm). From the comparison of the data, when comparing **Co_1_Fe_1_(II)-MOF-74@1** obtained by the one-step synthesis method with **Co_1_Fe_1_(II)-MOF-74@2** prepared by the template-guided method, the former has a relatively larger average particle size, with a difference of approximately 2.38 nm. This indicates that the template-guided method may have a certain influence on the control of particle size during the preparation process of this type of MOF. This may be attributed to the different reaction mechanisms of the two synthesis methods. The one-step synthesis method may lack effective limiting factors during the crystal growth process. In contrast, the template-guided method may, by virtue of the spatial confinement or guiding effect of the template, restrict the growth rate and growth direction of the crystals, thereby obtaining a relatively smaller and more uniform particle size. In addition, Figures S6 and S7 display, intuitively and clearly, the EDS element mapping distribution of the two MOFs, namely **Co_1_Fe_1_(II)-MOF-74@2** and **Co_1_Fe_1_(III)-MOF-74@2**, as well as the atomic proportion of Co/Fe.

**Figure 4:**
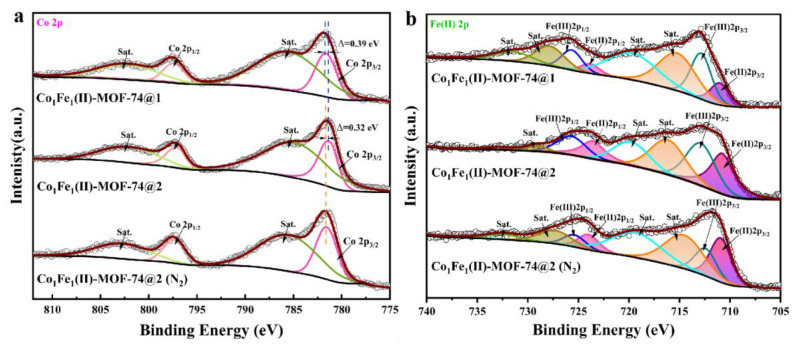
(**a**) High-resolution Co 2p XPS spectra of the three MOFs; (**b**) Fe(II) 2p XPS spectra of the three MOFs.

High-resolution X-ray photoelectron spectroscopy (XPS) was used to efficiently compare the chemical states and compositions of the elements in the four MOFs as well as in **Co_1_Fe_1_(II)-MOF-74@2 (N_2_)**, which is prepared under the protection of nitrogen flow. The full XPS spectra of the five samples (Figure S8) show the presence of the elements C, O, Co, and Fe and the semi-quantification of these elements, in agreement with the results of the EDS elemental analyses. High-resolution comparative XPS spectra of Co 2p and Fe 2p for three Fe-containing divalent-state MOFs are presented in Figure 4 (the splitting of orbital energy levels which is caused by the coupling of the orbital and spin motions of electrons results in the appearance of corresponding satellite peaks, which are marked with “sat.” in the spectrum). As can be clearly seen from the fine spectrum of Co 2p in Figure 4a, the peaks presenting near 781 eV and 797 eV correspond to Co 2p_3/2_ and Co 2p_1/2_, respectively, which well characterize the electronic configuration of Co(II) 2p. Further observation of the data in the figure reveals that there is a significant difference between the three MOF samples at the Co 2p_3/2_ peak position. Specifically, **Co_1_Fe_1_(II)-MOF-74@2** has a 0.39 eV shift in the Co 2p_3/2_ peak compared to **Co_1_Fe_1_(II)-MOF-74@1**, and a 0.32 eV shift in the Co 2p_3/2_ peak compared to **Co_1_Fe_1_(II)-MOF-74@2 (N_2_)**. These differences are a good indication that differences in both the means of assembly and the assembly environment can have an effect on the binding energy position at Co 2p_3/2_. The most likely reason lies in the variation of synthesis conditions, resulting in a disparity in the actual doping stoichiometry ratio of cobalt and iron. The alteration of the doping stoichiometry ratio impacts the electron transfer environment around the Co metal center, further leading to a change in local electron density and prompting a corresponding shift in the position of the binding energy [50]. It is worth noting that, in the Fe(II) 2p high-resolution XPS spectra of Figure 4b, the peak shape of Fe(II) 2p_3/2_ is broadened and distorted in comparison with the standard divalent-iron spectral peaks in the database, indicating the possible presence of multiple splitting peaks [51]. Through split-encapsulation fitting analysis, the characteristic peaks of Fe(II) 2p_3/2_ and Fe(III) 2p_3/2_ were observed at 710.5 eV and near 711.5 eV, respectively, in the three samples of Fe^II^-containing MOFs, which indicates that the electronic configurations of both Fe(II) 2p and Fe(III) 2p are present simultaneously. This may be due to the fact that Fe^II^ is partially oxidized to Fe^III^ during the reaction process, thus building an environmental system where Fe^II^ and Fe^III^ coexist. Based on the XPS analysis data, the ratio of Fe^II^/Fe^III^ showed that **Co_1_Fe_1_(II)-MOF-74@1** (0.53) < **Co_1_Fe_1_(II)-MOF-74@2** (0.89) < **Co_1_Fe_1_(II)-MOF-74@2 (N_2_)** (1.72). This clearly shows that most of Fe^II^ is oxidized to Fe^III^ in MOFs prepared by one-step synthesis, while more Fe^II^ is retained in MOFs prepared under N_2_ atmosphere, which constitutes a good model for rationally regulating the valence state of the transition metal and the performance of OER. The phenomenon reveals a synergistic effect between the two different valence states in **Co_1_Fe_1_(II)-MOF-74**. This synergistic effect encompasses the change in valence electrons and the adjustment of the electronic properties of the active centers, resulting in the optimization of the adsorption/desorption of oxygen-containing substances (OH*, OOH*, and O*). Such optimization plays a crucial role in accelerating the catalytic process of the OER. In addition, the high-resolution XPS fine spectra of C 1s, O 1s, and Fe(III) 2p were characterized (refer to Figures S9 and S10 for details).

In **Co_x_Fe_y_-MOF-74**, the stoichiometric ratio of cobalt and iron, which are catalytically active metal elements, influences the quantity and performance of active sites directly. Next, the inductively coupled plasma–optical emission spectroscopy (ICP-OES) characterization technique was employed to verify the actual Co/Fe stoichiometric ratio in **Co_1_Fe_1_-MOF-74** when the feed ratio of Co to Fe was 1:1. The specific data are presented in Tables S1–S4. The test results showed that the actual stoichiometric Co/Fe ratio of the **Co_1_Fe_1_(II)-MOF-74@2** catalyst assembled by the template-directed method was 2.311:2.288, which was closest to 1:1. And the Co/Fe ratio of 2.176:1.809 for **Co_1_Fe_1_(III)-MOF-74@2** was the next closest. This is attributed to the fact that Co-MOF-1D serves as a precursor, providing a distinct binding site for the precise coordination of the secondary metal Fe ions and contributing to the ordered insertion of the metal ions. However, the Co/Fe ratios of the one-step synthesized **Co_1_Fe_1_(II)-MOF-74@1** and **Co_1_Fe_1_(III)-MOF-74@1** were 2.064:1.74 and 2.432:1.751 in turn. This result suggests that, in most of the oxygen-coordination-based MOF materials in a disordered state, the Co atoms are more prone to ligand binding for forming complexes.

In summary, by utilizing XRD, various spectroscopic techniques, and electron microscopy spectroscopy analysis, we have successfully synthesized ordered bimetallic **Co_1_Fe_1_(II)-MOF-74@2** and **Co_1_Fe_1_(III)-MOF-74@2**, and controlled the stoichiometric ratios of the two metal sources, Co and Fe, to 1:1. Based on these results, we will further investigate the differences in the properties of one-step synthesis, ordered assemblies, and charge property modulation in the electrocatalytic OER, as indicated by the performance differences.

### 2.2. Electrochemical Performance Testing

**Figure 5:**
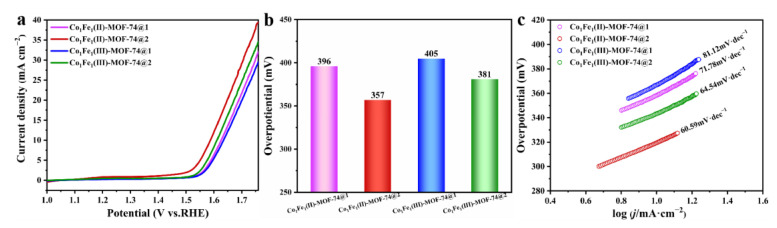
(**a**) LSV curve of four MOFs in 0.1M KOH; (**b**) comparison diagram of overpotential at current density of 10 mA cm^−2^; and (**c**) corresponding Tafel slope plot.

In order to ensure a single variable, the catalyst ink was prepared in the same manner, and the four MOF samples were successively loaded onto the same glassy carbon (GC) electrode with a maximum loading of approximately 0.36 mg·cm^−2^. The OER performance of the samples was tested by employing a typical three-electrode system with 0.1 M KOH solution as the alkaline electrolyte. As shown in Figure 5a, at a voltage of 1.6 V, **Co_1_Fe_1_(II)-MOF-74@2** exhibits the highest current density, with **Co_1_Fe_1_(III)-MOF-74@2** following. Figure 5b clearly presents the overpotentials of the four catalyst samples at a current density of 10 mA·cm^−2^. The overpotential of the **Co_1_Fe_1_(II)-MOF-74@2** catalyst is the smallest, being 357 mV. The Tafel slope can reveal the mechanism and kinetic characteristics of the electrocatalytic oxygen evolution reaction process. It is obtained by linearly fitting the Tafel equation to the linear scanning voltammetry (LSV) curve data, as shown in Figure 5c. The results are as follows: **Co_1_Fe_1_(II)-MOF-74@2** (60.59 mV·dec^−1^) < **Co_1_Fe_1_(III)-MOF-74@2** (64.54 mV·dec^−1^) < **Co_1_Fe_1_(II)-MOF-74@1** (71.78 mV·dec^−1^) < **Co_1_Fe_1_(III)-MOF-74@1** (81.12 mV·dec^−1^). This indicates that **Co_1_Fe_1_(II)-MOF-74@2** can drive the reaction to proceed at a lower applied voltage and has faster reaction kinetics compared with the other three MOFs. This excellent performance is attributed to the unique synergistic mechanism between Co and Fe metals. In this catalytic system, Co atoms serve as the central active sites, strongly adsorbing reactant molecules. The Fe atoms are located adjacent to the Co atoms. When the electron cloud of the reactants changes due to the adsorption by Co, the Fe atoms can respond promptly, adjusting their own electronic states and constructing complementary electron transfer channels [38]. Plus, in the structure of this MOF, the orderly arrangement of Co and Fe enables the reactant molecules to be adsorbed, transformed, and desorbed orderly between the two, reducing the diffusion hindrance on the catalyst surface and optimizing the reaction kinetics [52]. In summary, the above-mentioned test results suggest that the synergistic effect between metals can be exploited to a greater extent by means of the precise and orderly doping assembly method, thereby exhibiting more outstanding OER performance characteristics.

**Figure 6:**
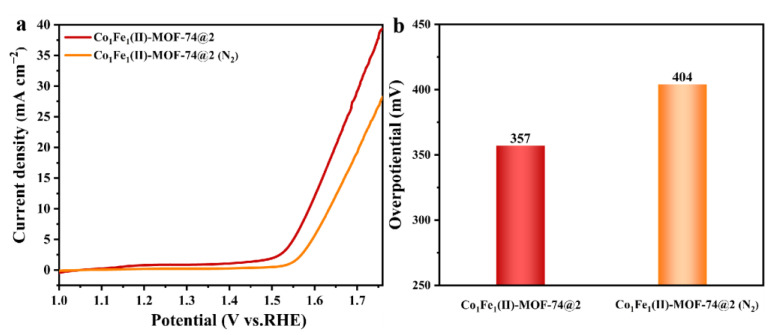
(**a**) LSV curves of Co_1_Fe_1_(II)-MOF-74@2 and Co_1_Fe_1_(II)-MOF-74@2 (N_2_); (**b**) overpotential of **Co_1_Fe_1_(II)-MOF-74@2** and **Co_1_Fe_1_(II)-MOF-74@2 (N_2_)** at current density of 10 mA cm^−2^.

The above OER performance test results also show that **Co_1_Fe_1_(II)-MOF-74@1** has better performance than **Co_1_Fe_1_(III)-MOF-74@1**, and **Co_1_Fe_1_(II)-MOF-74@2** likewise outperforms **Co_1_Fe_1_(III)-MOF-74@2**. It is evident that the valence state of iron has an effect on the performance of the OER. To deeply explore the mechanism of how valence differences influence the OER properties, in this study, a sample of **Co_1_Fe_1_(II)-MOF-74@2**, labeled as **Co_1_Fe_1_(II)-MOF-74@2 (N_2_)**, was re-prepared using the template-directed method under a nitrogen flow atmosphere (with other conditions unchanged). The electrocatalytic OER performance was tested using the same method. It can be seen from the LSV polarization curves in Figure 6a and the overpotential comparison plot in Figure 6b that there is a difference in OER performance between **Co_1_Fe_1_(II)-MOF-74@2** and **Co_1_Fe_1_(II)-MOF-74@2 (N_2_)**. The reason is that the following reaction is carried out in the air atmosphere at the same time, 4Fe^2+^ + O_2_ + H_2_O = 4Fe^3+^ + 4OH^−^, so that part of Fe^II^ is converted to Fe^III^, forming a bimetallic MOF catalyst with a mixed valence of metallic iron elements, which is consistent with the coexistence of Fe^II^ and Fe^III^ in the above Fe 2p high-resolution XPS spectrum. Metallic elements with different valence states typically possess distinct electronic structures and redox capacities. These differences affect their adsorption and activation abilities towards reactants, thereby leading to changes in their catalytic activities [53]. In the **Co_1_Fe_1_(II)-MOF-74@2** catalyst, the synergistic effect resulting from the coexistence of Fe^II^ and Fe^III^ in the mixed-valence state enhances the OER performance of this catalyst. Therefore, within the **Co_1_Fe_1_(II)-MOF-74@2** catalyst, the synergistic effect arising from the coexistence of the mixed-valence states of Fe^II^ and Fe^III^, in conjunction with that between Co and Fe metals, jointly enhances the OER performance of this catalyst. This phenomenon undoubtedly reveals that the valence modulation of metal ions also has a crucial impact on the electrocatalytic OER process.

**Figure 7:**
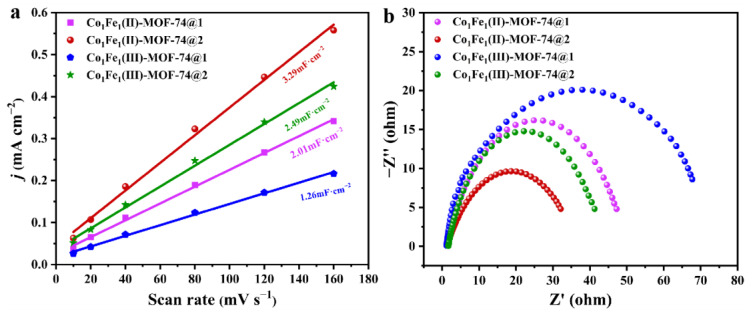
(**a**) Double-layer capacitance diagram of four MOFs; (**b**) electrochemical impedance diagrams (EIS) of four MOFs.

The electrochemically active area (ECSA) reflects the effective area of the catalyst that actually participates in the electrochemical reaction [54]. This is crucial for evaluating the activity of the catalyst. In general, a larger ECSA indicates more active sites on the catalyst surface that can participate in electrochemistry and, consequently, a higher catalytic activity. In this paper, cyclic voltammetry (CV, Figure S11) is employed to measure the current response in the non-Faraday potential region, and the bilayer capacitance *C*_dl_ is calculated by linearly fitting the slope, which serves as a means of evaluating the ECSA of the four MOF samples. The test results, as shown in Figure 7a, indicate that the *C*_dl_ of **Co_1_Fe_1_(II)-MOF-74@2** (3.29 mF·cm^−2^) is significantly higher than that of the other three MOF catalysts, with **Co_1_Fe_1_(III)-MOF-74@2** (2.49 mF·cm^−2^) following. Since the ECSA is proportional to the *C*_dl_ value [55], this implies that bimetallic MOFs with two-step ordered assembly and mixed-valence states can expose more active sites and exhibit higher catalytic activity when used as alkaline OER electrocatalysts compared to one-step synthesized and single-valence bimetallic MOFs. In addition, electrochemical impedance spectroscopy (EIS) was utilized to probe the charge transfer dynamics of the catalytic reactions of the four MOF materials, as shown in Figure 7b. From this Nyquist plot, it can be observed that **Co_1_Fe_1_(II)-MOF-74@2** with a mixed-valence state displays a smaller semicircle diameter of the impedance spectrum compared to the other three MOFs, which suggests that it has a lower charge transfer resistance (Rct) and a faster charge transfer rate, which can promote the OER more efficiently [56]. This result is in line with the previous LSV curve results and further corroborates the potential of the template-directed method of ordered assembly and valence modulation in enhancing the performance of the electrocatalytic OER.

**Figure 8:**
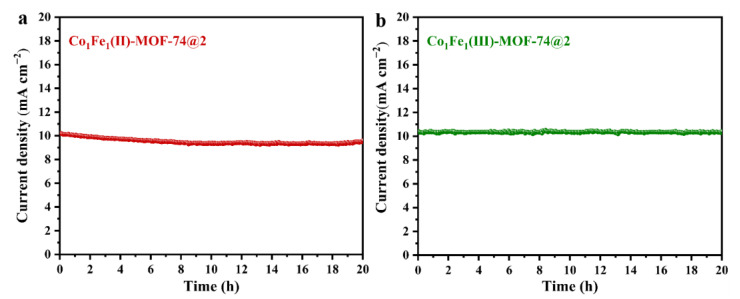
The 20 h chronocurrent curve was obtained at constant potential: (**a**) **Co_1_Fe_1_(II)-MOF-74@2**; (**b**) **Co_1_Fe_1_(III)-MOF-74@2**.

For each electrocatalytic reaction, enhancing the catalytic activity of the catalyst while ensuring its stability during the reaction process is a crucial point for realizing the commercial application of the catalyst. In this paper, an in-depth analysis of the variation in current with time under constant potential conditions has been conducted for two catalysts, namely **Co_1_Fe_1_(II)-MOF-74@2** and **Co_1_Fe_1_(III)-MOF-74@2**, which were prepared by the template-directed ordered assembly method. The test results are shown in Figure 8. As can be observed from this figure, the current densities of the two MOFs in a 0.1 M KOH solution did not exhibit any significant fluctuations and remained in a relatively stable state during the 20 h test period. In addition, Figure S12 shows the comparison of XRD patterns of the two MOFs before and after the OER. The original peak shapes are basically maintained, which once again confirms the good stability of the samples during the OER test. This result provides strong support and a solid basis for the potential practical applications of these two MOFs.

## 3. Materials and Methods

### 3.1. Materials

2,5-dihydroxyterephthalic acid (H_4_dobdc), cobalt(II) acetate tetrahydrate (Co(CH_3_COO)_2_·4H_2_O), Iron(II) acetate (Fe(CH_3_COO)_2_), hydroxydiacetyl iron hydrate (Fe(OH)(CH_3_COO)_2_·nH_2_O), and Nafion (5 wt%) were purchased from Shanghai Aladdin Biochemical Technology Co., Ltd., (Shanghai, China) N,N-Dimethylfumarate (DMF), KOH, and EtOH were from Sinopharm Chemical Reagent Co., Ltd., (Shanghai, China) All aqueous solutions were prepared with DI water. All chemicals were analytical grade and used as received without further purification.

### 3.2. Synthesis Methods

**Co-MOF-1D**. Prepared this by the mechanical ball milling method. Firstly, weigh Co(CH_3_COO)_2_·4H_2_O (0.5 mmol, 0.1245 g) and H_4_dobdc (0.5 mmol, 0.099 g) and pour them into an onyx ball milling jar with stirring and mixing, followed by the addition of 80 µL of deionized water, and set the speed of the ball mill to 500 rpm for a milling process of 2 h. Air dry the fume hood to finally obtain a pink-colored product Co-MOF-1D.

**Co_1_Fe_1_(II)-MOF-74@2**. Configure 14 mL of DMF:EtOH:H_2_O = 12:1:1 mixed solvent into a 50 mL explosion-proof single-necked round-bottom flask. Pour the synthesized **Co-MOF-1D** (0.55 mmol, 200 mg) into it and stir evenly. Then add Fe(CH_3_COO)_2_ (0.55 mmol, 96 mg) as the secondary metal into the above-mixed solution. Heat it to 120 °C under oil bath conditions maintain a rotation speed of 200 revolutions per minute, and continuously stir for 5 h. After that, wash the obtained product with ethanol at least three times, then filter it, place it in a vacuum oven and dry it at 60 °C for 24 h.

**Co_1_Fe_1_(III)-MOF-74@2**. The preparation steps of **Co_1_Fe_1_(III)-MOF-74@2** are similar to those of **Co_1_Fe_1_(II)-MOF-74@2**, only replace Fe(CH_3_COO)_2_ (0.55 mmol, 96 mg) with Fe(OH)(CH_3_COO)_2_·nH_2_O (0.55 mmol, 107 mg) as the secondary metal. The obtained product is also washed with ethanol, filtered, and dried in a vacuum oven at 60 °C for 24 h.

**Co_1_Fe_1_(II)-MOF-74@2 (N_2_)**. Configure 14 mL of DMF:EtOH:H_2_O = 12:1:1 mixed solvent into a 50 mL explosion-proof three-necked round-bottom flask. Pass nitrogen and stir with a magnet for 15 min. Add the synthesized **Co-MOF-1D** (0.55 mmol, 200 mg) into it, and then mix in Fe(CH_3_COO)_2_ (0.55 mmol, 96 mg). Under a nitrogen atmosphere, heat in an oil bath at 120 °C for 5 h. After that, wash the obtained product with ethanol at least three times, then filter it, and place it in a vacuum oven and dry it at 60 °C for 24 h.

**Co_1_Fe_1_(II)/(III)-MOF-74@1**. Weigh H_4_dobdc (2 mmol, 0.396 g), Co(CH_3_COO)_2_·4H_2_O (1 mmol, 0.249 g), and Fe(CH_3_COO)_2_ (1 mmol, 174 mg). Next, prepare a mixed solvent with a volume ratio of DMF:EtOH:H_2_O = 12:1:1. Add the weighed compounds in sequence to this mixed solvent and then ultrasonically treat the mixture for 10 min. Then, transfer the treated mixture to a stainless steel autoclave with a maximum capacity of 20 mL and a polytetrafluoroethylene lining. Keep it at a temperature of 120 °C for 12 h. Similarly, the preparation method of **Co_1_Fe_1_(III)-MOF-74@1** is similar, just replace Fe(CH_3_COO)_2_ (1 mmol, 174 mg) with Fe(OH)(CH_3_COO)_2_·nH_2_O (1 mmol, 194 mg). Finally, wash the obtained product with ethanol at least three times, then filter it, place it in a vacuum oven, and dry it at 60 °C for 24 h.

### 3.3. Characterization of Catalysts

The morphology and structure of the MOFs were analyzed by scanning electron microscopy (SEM, Hitachi S-4800, Hitachi High-Tech America, Inc., Tokyo, Japan) with energy dispersive spectroscopy (EDS). Infrared spectra were carried out by a THERMO NicoletNexus 470 FT-IR spectrometer (Thermo/Nicolet, Waltham, MA, USA). An XRD-7000 was used to collect the X-ray diffraction (XRD) patterns with a Cu Kα radiation source, which is produced by SHIMADZU (Kyoto, Japan). Thermogravimetric analysis (TGA) was conducted by a NETZSCH SAT-409PC (Netzsch-Gerätebau GmbH, Selb, Germany) in a nitrogen flow. The content of transition metals such as Co, Ni, and Fe was measured using X-ray photoelectron spectroscopy (XPS, THERMO ESCALAB 250Xi, Thermo, Waltham, MA, USA) and an Inductively Coupled Plasma Spectrometer (ICP, Agilent ICP-OES720, Aglient, Santa Clara, CA, USA).

### 3.4. Electrochemical Measurement

Electrochemical measurements were implemented in a three-electrode system with a Ag/AgCl (0.1 M KOH) electrode as the reference electrode and a carbon rod as the counter electrode; a glassy carbon (GC) electrode loaded with MOFs was used as the working electrode. All potentials were calculated by the Nernst equation (*E* (V vs. RHE) = *E* (V vs. Ag/AgCl) + 0.197 + 0.0592 pH), using the reversible hydrogen electrode (RHE) as a reference. The catalyst ink was prepared by the following method: 12 mg of catalyst and 12 mg conductive carbon powder were fully ground in a mortar, and then 5 mg of the above mixed powder was taken into a 2 mL centrifugation tube; 375 µL EtOH, 125 µL H2O, and 40 µL Nafion solution was added and ultrasonication was carried out for 60 min. An amount of 5 µL of homogeneous solution was spread evenly on a freshly polished glassy carbon electrode and it was placed in an infrared drying oven for complete drying. The catalyst mass loading was approximately 0.36 mg cm^−2^. The linear scan voltammetry (LSV) curves of obtained MOFs was recorded with an applied potential window of 0–0.8 V vs. Ag/AgCl; the scan rate was 5 mV s^−1^. From the cyclic voltammograms curves with the potential window 0–0.1 V vs. Ag/AgCl at different scan rates (v = 10, 20, 40, 80 120 and 160 mV s^−1^), we calculated the electrochemical double-layer capacitance (*C*_dl_) to obtain the electrochemically active surface area (ECSA). The long-term chronoamperometry test was practiced at an overpotential of current density of 10 mA cm^−2^. Electrochemical impedance spectroscopy (EIS) was recorded with the applied potential of 0.65 V versus RHE, and the frequency scan range was from 10^−2^ Hz to 10^5^ Hz.

Calculations. All potentials were corrected to the reversible hydrogen electrode (RHE), calculated by the Nernst equation:

*E* (V vs. RHE) = *E* (V vs. Ag/AgCl) + 0.197 V + 0.0592 × pH (1)

The Tafel slope was calculated by using the following Equation.

(2) η=blogj+a

where η is the overpotential (V), *j* is the current density (mA cm^−2^), and *b* is the Tafel slope (mV dec^−1^).

## 4. Conclusions

In this paper, **Co-MOF-1D** with a well-defined binding site was preliminarily synthesized by mechanical ball milling. Using this as a template to dope Fe ions, the ordered bimetallic **Co_1_Fe_1_-MOF-74@2** was successfully assembled, and the stoichiometric ratio of the two metal sources, cobalt and iron, was close to 1:1. Compared with the conventional one-step synthesis method, this bimetallic **Co_1_Fe_1_-MOF-74@2** assembled by template-directed assembly exhibits superior electrocatalytic performance. Meanwhile, the valence states of iron elements (Fe^II^ and Fe^III^) in the MOF were modulated, and the electrocatalytic materials **Co_1_Fe_1_(II)-MOF-74@2** and **Co_1_Fe_1_(III)-MOF-74@2** were prepared for investigating the effects of different valence states in MOF catalysts. The OER test results show that **Co_1_Fe_1_(II)-MOF-74@2** performs better, and this unique phenomenon is closely related to the gradual increment in the Fe ion valence state in this MOF, which contributes to the continuous enhancement of its performance before reaching the optimal steady state until the mixed-valence state reaches the optimal state. From the perspective of industrial application potential, this joint improvement in the performance of MOF catalysts through template-directed assembly, bimetallic synergism, and valence modulation has broad research potential. In water electrolysis for hydrogen production, it can reduce the overpotential of oxygen evolution, improve the efficiency of hydrogen production from electrical energy, and promote large-scale hydrogen production. In metal–air batteries, it can improve the charging and discharging performance and extend the service life, thus facilitating its application in electric vehicles. In industrial wastewater treatment, it can accelerate the oxidative decomposition of pollutants, improve the efficiency, and reduce the costs. In conclusion, template-directed assembly, intermetallic synergism, and valence modulation jointly enhance the electrocatalytic performance of MOFs, providing a research direction for the targeted synthesis and optimization of highly efficient catalysts.

## Data Availability

Data will be made available on request.

## Supplementary Materials

The following supporting information can be downloaded at https://www.mdpi.com/article/10.3390/molecules29245845/s1: Figure S1. XRD patterns of **Co_1_Fe_1_(**II**)-MOF-74@2 (N_2_)**. Figure S2. FT-IR spectrum of (a) four MOFs; (b) **Co_1_Fe_1_(**II**)-MOF-74@2** and **Co_1_Fe_1_(**II**)-MOF-74@2 (N_2_)**. Figure S3. TGA analysis of four MOFs. Figure S4. SEM images of four MOFs. Figure S5. Average particle size diagrams of four MOFs captured by scanning electron microscopy. Figure S6. SEM-EDS stratification and corresponding elemental mapping. Figure S7. EDS Elemental Analysis. Figure S8. Full XPS scan spectra of the five MOFs. Figure S9. (a) High-resolution C 1s XPS spectra of the four MOFs; (b) O 1s XPS spectra of the four MOFs. Figure S10. Fe(III) 2p XPS spectra of the two MOFs. Figure S11. CV curves of four MOFs. Figure S12. Comparison of XRD patterns of the catalyst before and after the OER reaction. Table S1. ICP analysis for **Co_1_Fe_1_(**II**)-MOF-74@1** (Co/Fe=2.064/1.747). Table S2. ICP analysis for **Co_1_Fe_1_(**II**)-MOF-74@2** (Co/Fe=2.311/2.288). Table S3. ICP analysis for **Co_1_Fe_1_(**III**)-MOF-74@1** (Co/Fe=2.432/1.751). Table S4. ICP analysis for **Co_1_Fe_1_(**III**)-MOF-74@2** (Co/Fe=2.176/1.809). Table S5. OER performance comparison of **Co_1_Fe_1_(**II**)-MOF-74@2**, **Co_1_Fe_1_(**III**)-MOF-74@2** and other reported electrocatalysts on a glassy carbon electrode or carbon cloth. It includes XRD patterns, SEM images, SEM-Mapping images, XPS spectra, TGA curves, FT-IR spectra, CV curves, ICP-OES analysis result tables and other charts and graphs [50,57,58,59,60,61,62,63,64,65,66,67,68,69,70,71].
