# Supplementary material for: Assembly and Valence Modulation of Ordered Bimetallic MOFs for Highly Efficient Electrocatalytic Water Oxidation
Source: Molecules. 2024 Dec 11;29(24):5845. doi: 10.3390/molecules29245845 (PMC11728488; doi:10.3390/molecules29245845)
Supplement: Supplementary file 1 [file molecules-29-05845-s001.zip › molecules-3334784-supplementary.pdf]

Supporting information

Assembly and valence modulation of ordered bimetallic MOFs for highly efficient electrocatalytic water oxidation

Yaling Wu,[1,2] Zhaopeng Sun,[2] Yingying Chen,[2] Dan Liu,[2] Yan Meng[1,*] and Zheng Yan[2,*]

1. Anhui Provincial Key Laboratory of Advanced Catalysis and Energy Materials, Anqing Normal University, Anqing 246133, P. R. China

2. College of Biological, Chemical Sciences and Engineering, Jiaxing University, Jiaxing 314001, P. R. China

* Correspondence to: mengyan@aqnu.edu.cn (Y. Meng); yzheng158@zjxu.edu.cn (Z. Yan)

Contents:

1. Figures

S1. XRD patterns of **Co_1Fe_1(II)-MOF-74@2 (N_2)**.

S2. FT-IR spectrum of (a) four MOFs; (b) **Co_1Fe_1(II)-MOF-74@2** and **Co_1Fe_1(II)-MOF-74@2 (N_2)**.

S3. TGA analysis of four MOFs.

S4. SEM images of four MOFs.

S5. Average particle size diagrams of four MOFs captured by scanning electron microscopy.

S6. SEM-EDS stratification and corresponding elemental mapping.

S7. EDS Elemental Analysis.

S8. Full XPS scan spectra of the five MOFs.

S9. (a) High-resolution C 1s XPS spectra of the four MOFs;(b) O 1s XPS spectra of the four MOFs.

S10. Fe(III) 2p XPS spectra of the two MOFs.

S11. CV curves of four MOFs.

S12. Comparison of XRD patterns of the catalyst before and after the OER reaction.

2. Table

Table S1: ICP analysis for **Co_1Fe_1(II)-MOF-74@1**.

Table S2: ICP analysis for **Co_1Fe_1(II)-MOF-74@2** (Co/Fe=2.311/2.288).

Table S3: ICP analysis for **Co_1Fe_1(III)-MOF-74@1** (Co/Fe=2.432/1.751).

Table S4: ICP analysis for **Co_1Fe_1(III)-MOF-74@2** (Co/Fe=2.176/1.809).

Table S5: The comparison of OER performance between this work and other reported electrocatalysts on a glassy carbon electrode or carbon cloth.

3. References

Morphology characterization and Test results.

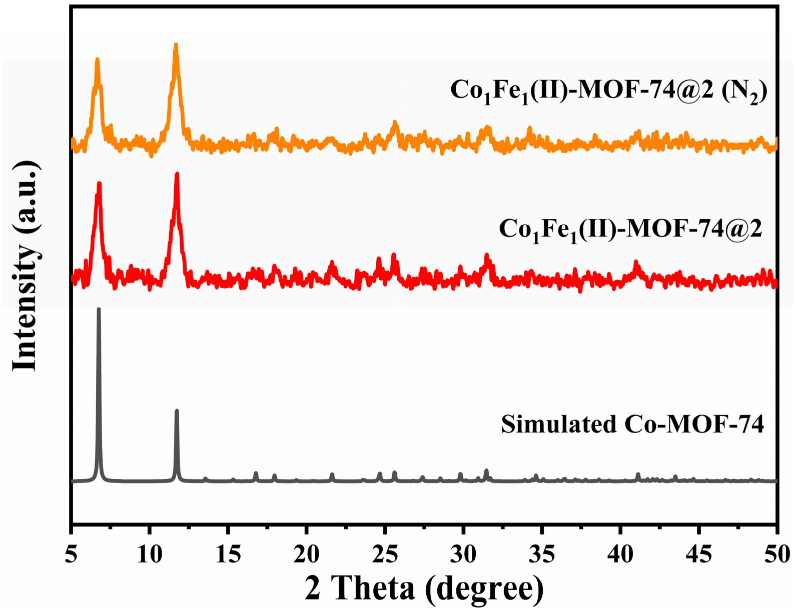

Figure S1. XRD patterns of **Co_1Fe_1(II)-MOF-74@2 (N_2)**.

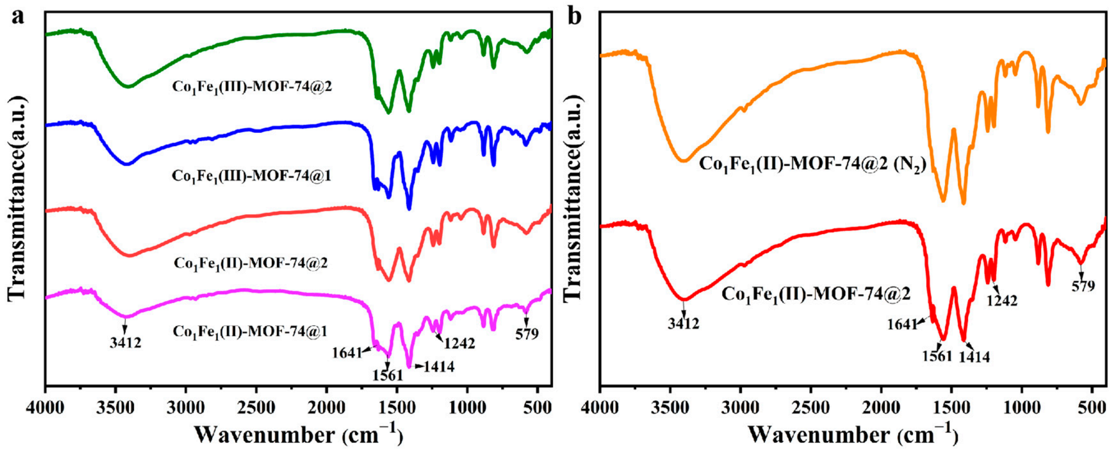

Figure S2. FT-IR spectrum of (a) four MOFs; (b) **Co_1Fe_1(II)-MOF-74@2** and **Co_1Fe_1(II)-MOF-74@2 (N_2)**.

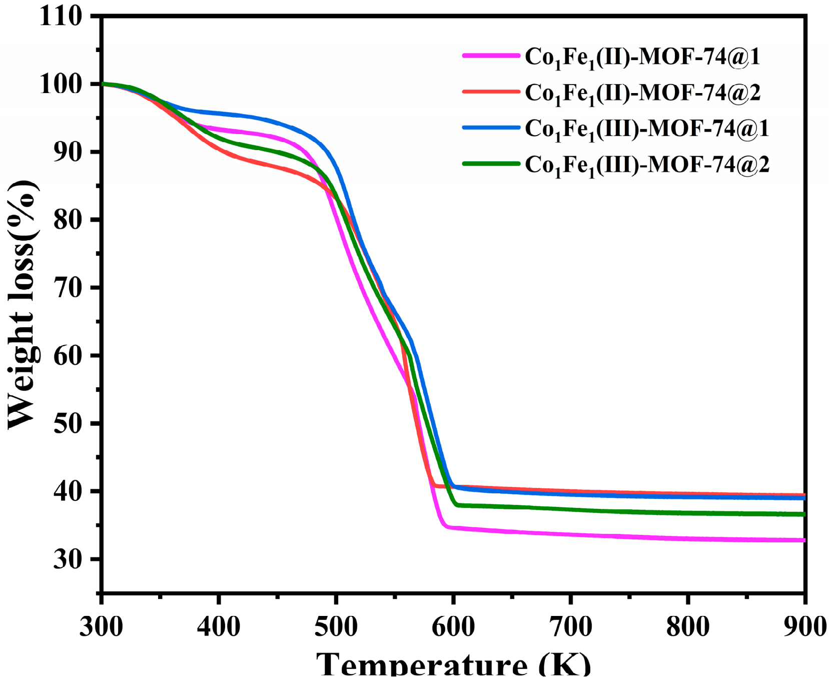

Figure S3. TGA analysis of four MOFs.

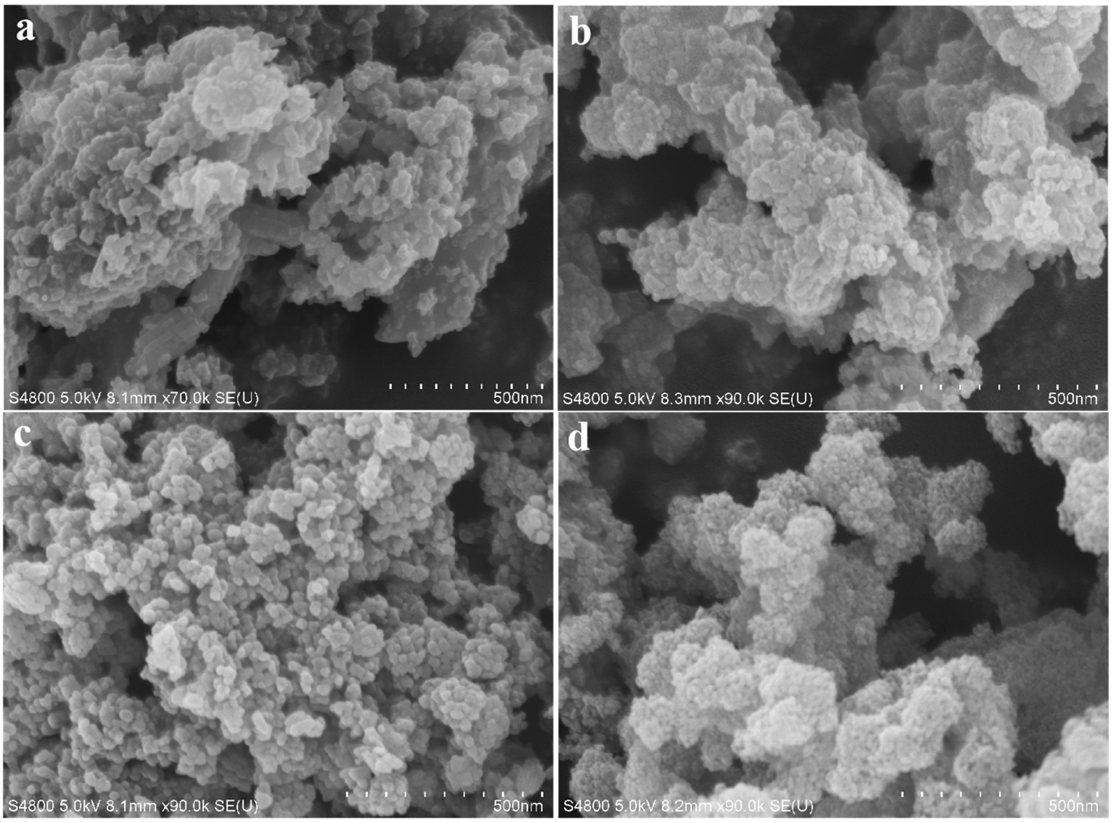

Figure S4. SEM images of (a) **Co_1Fe_1(II)-MOF-74@1**; (b) **Co_1Fe_1(II)-MOF-74@2**; (c) **Co_1Fe_1(III)-MOF-74@1**; (d) **Co_1Fe_1(III)-MOF-74@2**.

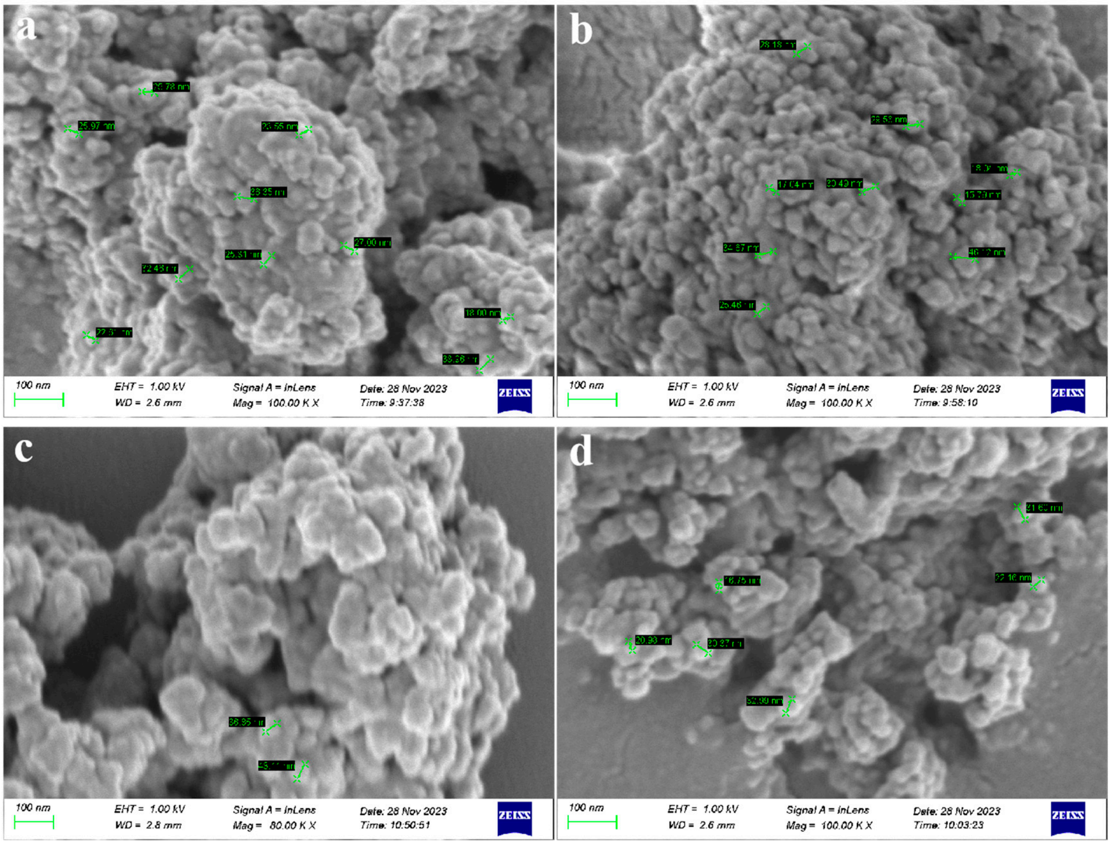

Figure S5. Average particle size diagrams of four MOFs captured by scanning electron microscopy: (a) **Co_1Fe_1(II)-MOF-74@1**; (b) **Co_1Fe_1(II)-MOF-74@2**; (c) **Co_1Fe_1(III)-MOF-74@1**; (d) **Co_1Fe_1(III)-MOF-74@2**.

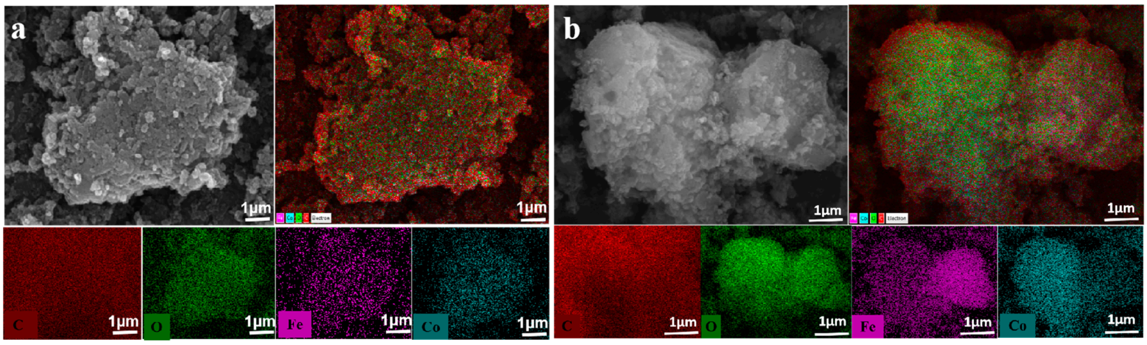

Figure S6. SEM, EDS stratification and corresponding elemental mapping of (a) **Co_1Fe_1(II)-MOF-74@2**; (b) **Co_1Fe_1(III)-MOF-74@2**.

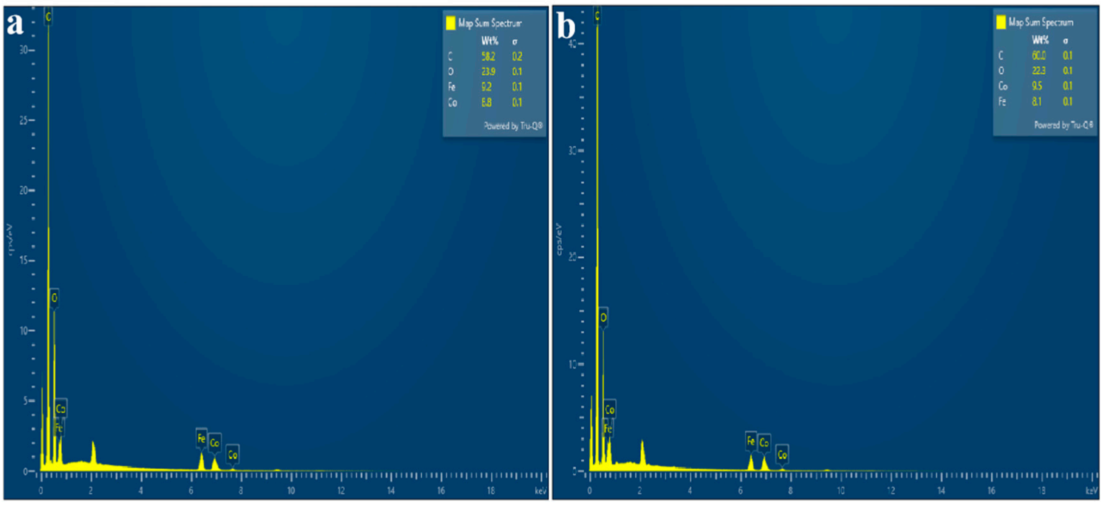

Figure S7. EDS Elemental Analysis of (a) **Co₁Fe₁(II)-MOF-74@2**,

(b) **Co₁Fe₁(III)-MOF-74@2**.

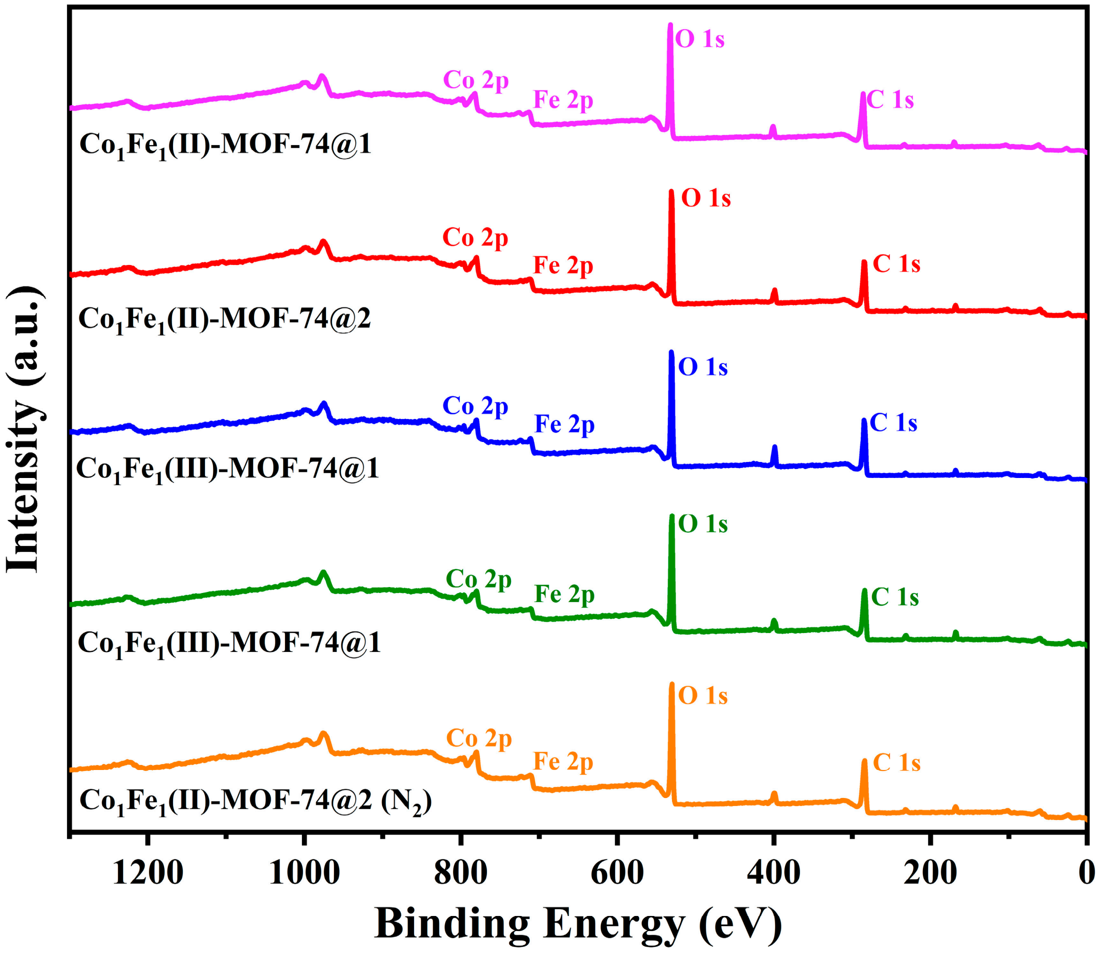

Figure S8. Full XPS scan spectra of the five MOFs.

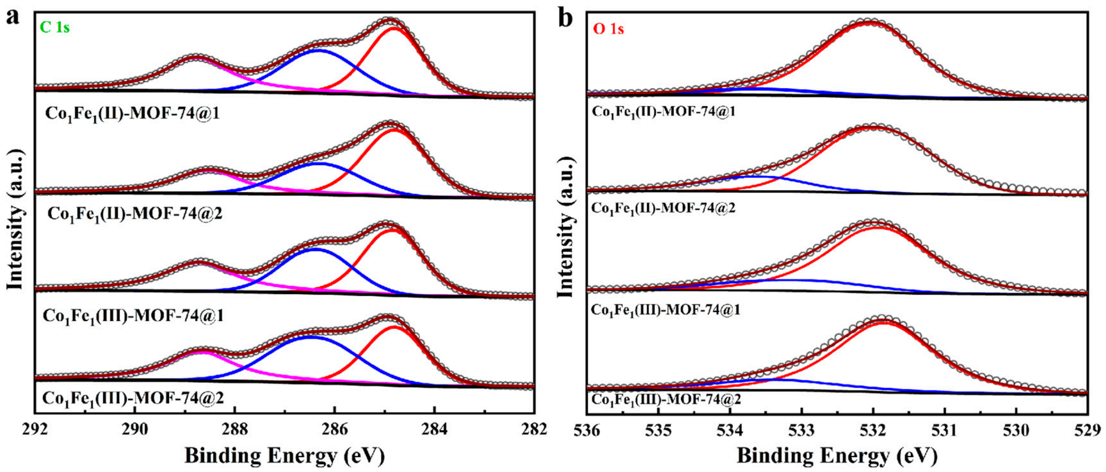

Figure S9. (a) High-resolution C 1s XPS spectra of the four MOFs; (b) O 1s XPS spectra of the four MOFs.

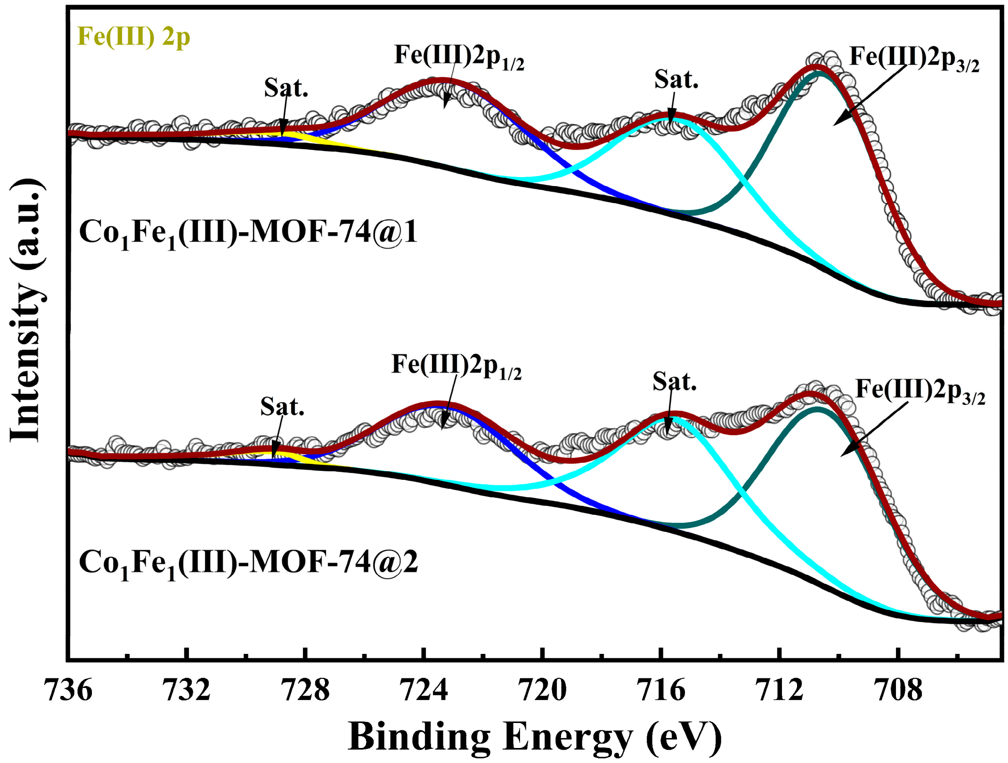

Figure S10. Fe(III) 2p XPS spectra of the two MOFs.

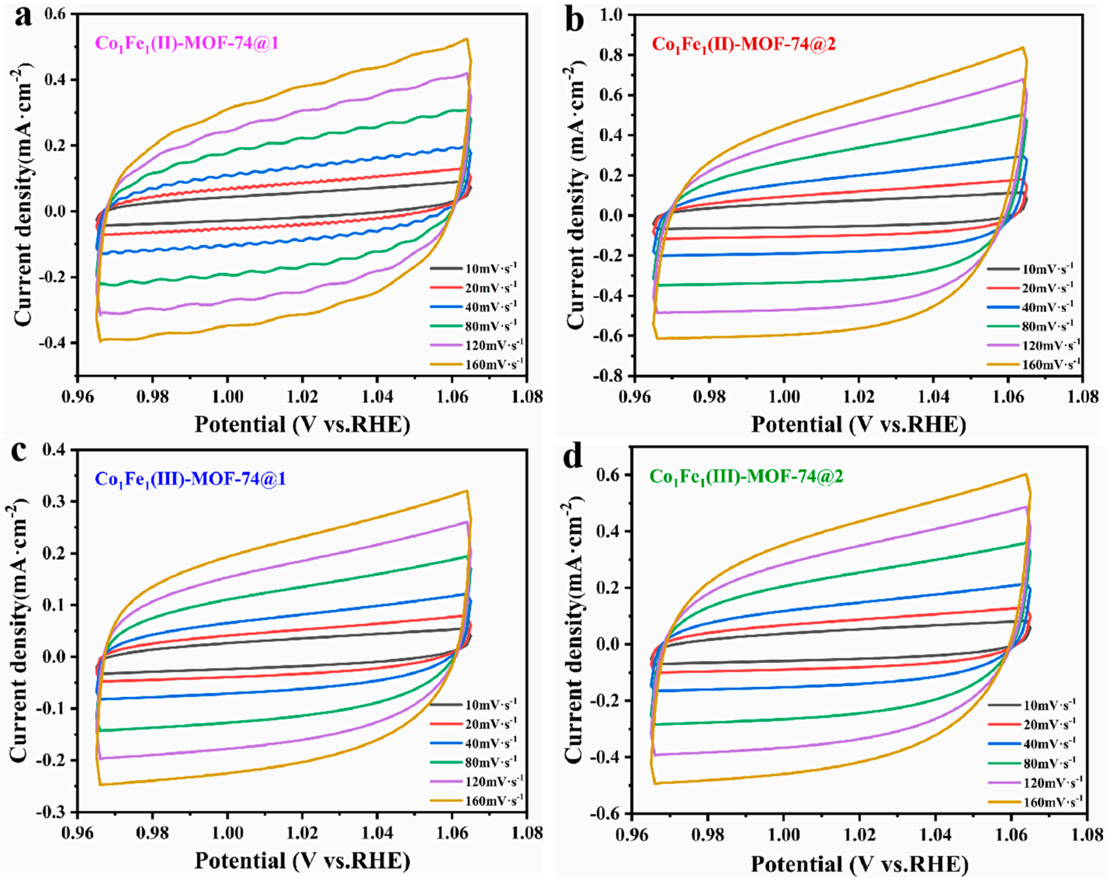

Figure S11. CV curves of four MOFs.

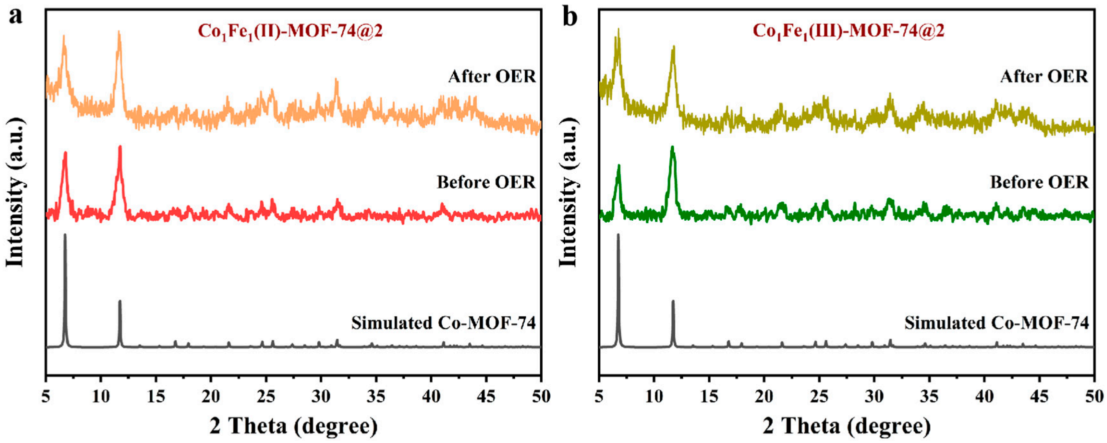

Figure S12. Comparison of XRD patterns of the catalyst before and after the OER reaction:

(a) **Co_1Fe_1(II)-MOF-74@2**, (b) **Co_1Fe_1(III)-MOF-74@2**

Table S1: ICP analysis for **Co_1Fe_1(II)-MOF-74@1** (Co/Fe=2.064/1.747).

**Co_1Fe_1(II)-MOF-74@1**	Sampling quality (g)	Volume (mL)	Coefficient of dilution	Instrument readings (mg/L)	Molar ratio
Co	0.0441	25	50	4.2882	2.064
Fe	0.0441	25	50	3.4511	1.747

Table S2: ICP analysis for **Co_1Fe_1(II)-MOF-74@2** (Co/Fe=2.311/2.288).

**Co_1Fe_1(II)-MOF-74@2**	Sampling quality (g)	Volume (mL)	Coefficient of dilution	Instrument readings (mg/L)	Molar ratio
Co	0.0431	25	50	4.6968	2.311
Fe	0.0431	25	50	4.4069	2.288

Table S3: ICP analysis for **Co_1Fe_1(III)-MOF-74@1** (Co/Fe=2.432/1.751).

**Co_1Fe_1(III)-MOF-74@1**	Sampling quality (g)	Volume (mL)	Coefficient of dilution	Instrument readings (mg/L)	Molar ratio
Co	0.0448	25	50	5.1366	2.432
Fe	0.0448	25	50	3.5059	1.751

Table S4: ICP analysis for **Co_1Fe_1(III)-MOF-74@2** (Co/Fe=2.176/1.809).

**Co_1Fe_1(III)-MOF-74@2**	Sampling quality (g)	Volume (mL)	Coefficient of dilution	Instrument readings (mg/L)	Molar ratio
Co	0.0336	25	50	3.4476	2.176
Fe	0.0336	25	50	2.7157	1.809

Table S5. OER performance comparison of **Co_1Fe_1(II)-MOF-74@2**, **Co_1Fe_1(III)-MOF-74@2** and other reported electrocatalysts on a glassy carbon electrode or carbon cloth.

**Catalyst**	**Over potential @ 10 mA cm^{-2}**	Electrolyte	**Electrolyte**	**Ref.**
**Co4-Co-MOF/CNT**	357	1.0 M KOH	GC	[57]
Co_4O_4 frameworks	430	1.0 M NaOH	GC	[58]
Ni-MOF/carbon fiber	430	1.0 M KOH	CC	[59]
NiMn-MOF/carbon fiber	320	1.0 M KOH	CC	[59]
Ni MOF nanosheets	416	1.0 M KOH	GC	[60]
Co corrole polymers	340	0.1 M KOH	GC	[61]
Co corrole/CNT	440	1.0 M KOH	GC	[62]
Co porphyrin/CNT	390	1.0 M KOH	GC	[63]
Co porphyrin@ZIF-67	416	1.0 M KOH	GC	[64]
Fe_3-Co_2 MOF	283	0.1 M KOH	GC	[65]
$[Co^{II}TP(Co^{III}C)_2](OH)_2$	412	0.1 M KOH	GC	[66]
Co-COF-366	484	0.1 M KOH	GC	[67]
CoTAPP-PATA-COF	420	0.1 M KOH	GC	[67]
CoTAPP-BDTA-COF	470	0.1 M KOH	GC	[67]
FeCo-MNS-1.0	298	0.1 M KOH	GC	[68]
CoFe-PYZ	300	0.1 M KOH	GC	[68]
ZIF-62-(Co)-Fe-CC	335	0.1 M KOH	CC	[70]
Fe_2Co-BPTC	365	0.1 M KOH	CC	[71]
CoFe-MOF	355	0.1 M KOH	GC	[50]
**Co_1Fe_1-MOF-74@2**	**357**	0.1 M KOH	**GC**	**This work**
**Co_1Fe_1(III)-MOF-74@2**	**381**	0.1 M KOH	**GC**	**This work**

References

[57] Z. Z. Liang, G. J. Zhou, H. Tan, Y. H. Mou, J. L. Zhang, H. B. Guo, S. J. Yang, H. T. Lei, H. Q. Zheng, W. Zhang, H. P. Lin, R. Cao, *Adv. Mater*. **2024**, *36*, 2408094.

[58] A. I. Nguyen, K. M. Van Allsburg, M. W. Terban, M. Bajdich, J. Oktawiec, J. Amtawong, M. S. Ziegler, J. P. Dombrowski, K. V. Lakshmi, W. S. Drisdell, J. Yano, S. J. L. Billinge, T. D. Tilley, *Proc. Natl. Acad. Sci. U.S.A*. **2019**, *116*, 11630.

[59] W. Cheng, X. F. Lu, D. Luan, X. W. Lou, *Angew. Chem. Int. Ed*. **2020**, *59*, 18234.

[60] P. Wu, S. Geng, X. Wang, X. Zhang, H. Li, L. Zhang, Y. Shen, B. Zha, S. Zhang, F. Huo, W. Zhang, *Angew. Chem. Int. Ed*. **2024**, *63*, e202402969.

[61] H. Lei, Q. Zhang, Z. Liang, H. Guo, Y. Wang, H. Lv, X. Li, W. Zhang, U.-P. Apfel, R. Cao, *Angew. Chem. Int. Ed*. **2022**, *61*, e202201104.

[62] X. Li, H. Qin, J. Han, X. Jin, Y. Xu, S. Yang, W. Zhang, R. Cao, *Adv. Funct. Mater*. **2024**, *34*, 2310820.

[63] H. Lv, X.-P. Zhang, K. Guo, J. Han, H. Guo, H. Lei, X. Li, W. Zhang, U.-P. Apfel, R. Cao, *Angew. Chem. Int. Ed*. **2023**, *62*, e202305938.

[64] Z. Liang, H. Guo, G. Zhou, K. Guo, B. Wang, H. Lei, W. Zhang, H. Zheng, U.-P. Apfel, R. Cao, *Angew. Chem. Int. Ed*. **2021**, *60*, 8472.

[65] J.-Q. Shen, P.-Q. Liao, D.-D. Zhou, C.-T. He, J.-X. Wu, W.-X. Zhang, J.-P. Zhang, X.-M. Chen, *J. Am. Chem. Soc*. **2017**, *139*, 1778.

[66] A. Aljabour, H. Awada, L. Song, H. Sun, S. Offenthaler, F. Yari, M. Bechmann, M. C. Scharber, W. Schöfberger, *Angew. Chem. Int. Ed*. **2023**, *62*, e202302208.

[67] M. Liu, S. Liu, C.-X. Cui, Q. Miao, Y. He, X. Li, Q. Xu, G. Zeng, *Angew. Chem. Int. Ed*. **2022**, *61*, e202213522.

[68] L. Zhuang, L. Ge, H. Liu, Z. Jiang, Y. Jia, Z. Li, D. Yang, R. K. Hocking, M. Li, L. Zhang, X. Wang, X. Yao, Z. Zhu, *Angew Chem Int Ed*, **2019**, *58,* 13565.

[69] R. Lin, X. Li, A. Krajnc, Z. Li, M. Li, W. Wang, L. Zhuang, S. Smart, Z. Zhu, D. Appadoo, J. R. Harmer, Z. Wang, A. G. Buzanich, S. Beyer, L. Wang, G. Mali, T. D. Bennett, V. Chen, J. Hou, *Angew Chem Int Ed*, **2022**, *61*: e202112880.

[70] X. L. Wang, L. Z. Dong, M. Qiao, Y. J. Tang, J. Liu, Y. Li, S. L. Li, J. X. Su, Y. Q. Lan, *Angew Chem Int Ed*, **2018**, *57*: 9660.

[71] P. Manna, J. Debgupta, S. Bose and S. K. Das, *Angew. Chem. Int. Ed*., **2016**, 55, 2425.
